# Competitive Conditions in Global Value Chain Networks: An Assessment Using Entropy and Network Analysis

**DOI:** 10.3390/e22101068

**Published:** 2020-09-23

**Authors:** Georgios Angelidis, Evangelos Ioannidis, Georgios Makris, Ioannis Antoniou, Nikos Varsakelis

**Affiliations:** School of Economics, Faculty of Economic and Political Sciences, Complex Systems Analysis Laboratory (COSAL), Laboratory of Economic Analysis and Policy (LEAP), Aristotle University of Thessaloniki, 541 24 Thessaloniki, Greece; angelidig@econ.auth.gr (G.A.); ioannidek@math.auth.gr (E.I.); geomak@math.auth.gr (G.M.); barsak@econ.auth.gr (N.V.)

**Keywords:** global value chain, complex networks, competitive conditions, entropy, degree, centralization

## Abstract

We investigated competitive conditions in global value chains (GVCs) for a period of fifteen years (2000–2014), focusing on sector structure, countries’ dominance and diversification. For this purpose, we used data from the World Input–Output Database (WIOD) and examined GVCs as weighted directed networks, where countries are the nodes and value added flows are the edges. We compared the in-and out-weighted degree centralization of the sectoral GVC networks in order to detect the most centralized, on the import or export side, respectively (oligopsonies and oligopolies). Moreover, we examined the in- and out-weighted degree centrality and the in- and out-weight entropy in order to determine whether dominant countries are also diversified. The empirical results reveal that diversification (entropy) and dominance (degree) are not correlated. Dominant countries (rich) become more dominant (richer). Diversification is not conditioned by competitiveness.

## 1. Introduction

National economies are becoming more and more interconnected and complex in an unprecedented way [1]. Some countries argue in favor of protectionism [2] as an economic policy to prevent economic loss, although protectionism was more relevant before the Second World War. World production and trade are increasingly structured around global value chains (GVCs) [3]. “Global value chains” (GVCs) incorporate the producers in global product markets [4] in terms of: (a) the activities required to bring a product or service from conception through the different phases of production delivery to final consumers, and final disposal after use [5]; (b) the value added of all activities that are directly and indirectly needed to produce a final product [6]; and (c) the full range of activities that firms undertake to bring a product or a service from its conception to its end use by final consumers [3]. Firms locate selected production stages in different countries, exploiting the comparative advantage of the selected countries. A country has a comparative advantage in a given industry when it is more productive in this industry compared to other countries [7]. Falling trade costs induce countries to specialize according to comparative advantage [8]. This strategy results in the emergence of countries with a dominant position in the relevant sectoral GVC network. The economies of the emergent dominant countries concentrate the biggest share of input or output value added within the sectoral GVC network. On the other hand, dominance is limiting competition within the sectoral network. GVC analysis highlights the patterns of international trade, production, and employment that determine the prospects for competition [9]. Unlike trade policies, which can be classified based on their impact on welfare, the value of various competition rules depends more on the objective of the political authorities [10]. As competition is a key political issue, decision makers should know:

*1*  Which sectoral GVC networks are highly centralized and what is the extent of centralization?
*2*  Which countries within a sectoral GVC network are highly diversified?
*3*  Are the dominant countries within a sectoral GVC network also highly diversified?

Network analysis has been recently applied on economic networks like GVCs for the examination of their structure, evolution, connectivity, and countries’ participation. Amador and Cabral [11] studied GVCs as weighted directed networks with countries as nodes and value added flows as edges, using WIOD (World Input–Output Database, Release 2013). They examined the evolution via the degree of centrality. Moreover, they analyzed [12] GVCs as six sub-networks: (a) a network of total foreign value added in goods exports; (b) a network of total foreign value added in services exports; (c) a network of goods with foreign value added in goods exports; (d) a network of goods with foreign value added in services exports; (e) a network of services with foreign value added in goods exports; and (f) a network of services with foreign value added in services exports. Cerina et al. [13] used the same database but explored GVCs as an interconnected network where the nodes are the industries and the edges are the value of goods flows between them. They detected key industries through assortativity, clustering coefficient, degree and Page Rank centrality. Cingolani et al. [14] used the BACI-CEPII (Base pour l’Analyse du Commerce International—Centre d’Etudes Prospectives d’Informations Internationales) database and examined the global value network centrality in order to find the country’s position at different production stages in the sectors “Electronics”, “Motor Vehicles”, “Textiles and Apparel” for the years 2007 and 2014. Criscuolo and Timmis [15] used OECD Inter-Country Input-Output (ICIO) Tables (1995–2011) and calculated Bonacich–Katz centrality on a network that nodes are country-sector units and edges are input flows in order to map key hubs, spokes and the periphery. Moreover, they [16] used cross-country firm-level data from ORBIS and examined how centrality affects the diffusion of productivity across firms, sectors and economies. Ferrantino and Taglioni examined [17] whether GVCs’ evolution contributed in trade slowdown after the “Trade Collapse of 2008–9”, using degree centrality, and discovered three main blocks of countries which appear polarized. Jouanjean studied [18] GVC participation and economic transformation computing backward centrality, forward centrality and eigenvector centrality. Shepherd [19] used the Eora database for the years 1996, 2001, 2006, 2011 and adopted eigen-centrality to measure the value chain connectivity for the two sectors (textiles and agriculture). Xing et al. [20] suggested new measurements using betweenness centrality in order to redefine the transfer route of intermediate goods in GVCs. Coquidé et al. [21] applied the reduced Google matrix (REGOMAX) algorithm on the United Nations COMTRADE database in order to analyze the multiproduct world trade in the years 2004–2016. They also applied REGOMAX [22] to the bitcoin network, where the nodes are the users and transactions are the edges. From PageRank and CheiRank probabilities, analogous to trade import and export, they specified the dimensionless trade balance of each user and modeled the contagion propagation on the network. The above work has not addressed the issue of the diversification of GVC networks. Entropy, being the natural tool for the analysis of diversification, has been employed as an indicator of incoming economic collapse [23].

The goal of our work is to address the above three questions, using entropy combined with network theory. We used a data set from WIOD, which covered 44 countries and 54 sectors for the examination period, 2000–2014, to construct 810 (54 sectors × 15 years) sectoral GVC networks. We computed in- and out-weight entropies as measures of the variety of value added flows among countries in GVC networks. We used in- and out-weighted degree centralization as a measure of the detection of limited competition at the country level in sectoral GVC networks. We did not address competition among firms. The high in-weighted degree centralization of a sectoral GVC network means that a few importers receive a high share of value added from the exporters, so they can put pressure on the trade prices and conditions (oligopsony), while low in-weighted degree centralization describes a sector with high competition where the imported share is more uniformly distributed to the importers. High out-weighted degree centralization in a sectoral GVC network means that a few exporters dominate the supply of value added which gives them the power to regulate prices (oligopoly), while low out-weighted degree centralization describes a sector with high competition where the exported share is more uniformly distributed to the exporters. Furthermore, we used entropy in order to examine if the value added share was imported /exported from/to many partners. Entropy rises with diversification, and contrariwise, declines with specialization [24,25]. Thus, if a dominant importer/exporter exhibits high in-/out-weight entropy, it is assumed that its value-added imports/exports are scattered to many partners, and consequently, it could be or become the regulator of the sectoral network, which harms the competition conditions.

Computing the evolution of entropy and the degree of dominant countries in the most and least centralized sectoral GVC networks, we evaluated their competition conditions and the possibility for change. The empirical results reveal that trade dominance is not interlaced with the diversification; the dominants are not necessarily the most diversified. Nevertheless, these two features belong to a few countries regardless of the centralization of the network. Hence, competitive conditions do not determine the diversification or the dominance. Furthermore, diversification remains stable on average but dominance is rising in the centralized sectoral GVC networks, a fact which indicates low competitive conditions contribute to maintaining dominance.

The data and methodology are presented in Section 2. Relevant concepts from network theory are presented in Section 3. The empirical findings of the network analysis and discussion are presented in Section 4 and our concluding remarks in Section 5.

## 2. Data and GVCs Network Construction

This empirical research uses data from the World Input–Output Database (WIOD)—2016 release. WIOD gathers some special features suitable for GVC studies, namely: (a) WIOD is appropriate for analysis over several years; (b) WIOD is derived from official and publicly available sources; (c) WIOD is based on national supply and use tables (SUT); (d) the construction of WIOD is based on transparent methods [26]; and (e) WIOD is publicly available and free (at http://www.wiod.org/release16). WIOD consists of a set of supply and use tables combined with data on international trade in goods and services. Gross trade flows are provided in current prices, denoted in millions of dollars among 28 EU countries, 15 other major countries and an extra area, named ROW (rest of the World), which embodies the remaining part of the world economy [27]. ROW participation is non-negligible. The 43 major economies include more than 85% of the world gross domestic product (in 2008). The remaining economies (less than 15%) are collectively estimated as ROW. Due to the relatively large size of the ROW, this part of the world economy cannot be ignored in analyses of global trade [26]. Therefore, it is important how ROW is estimated [28]. In the WIOD, ROW was estimated from the totals for industry output and final use categories from the UN National Accounts [26]. WIOD covers 54 sectors for the period from 2000 to 2014 and is widely adopted in empirical studies of GVCs [6,11,12,13,26,29]. Comparing WIOD to other databases, for example GTAP (Global Trade Analysis Project) Data Base, we can observe that the data in GTAP are not open, are not grounded in official statistics and are only available for certain benchmark years, which precludes the analyses of long-term trends [26]. To calculate the value added flows among countries for each sector, we followed Leontief’s decomposition technique which is widely adopted [26,28,30,31,32,33], implemented in R by the “Decompr” software [34].

We constructed one weighted directed network for each sector per year, as follows: the nodes represent the 44 countries and the edges (links) represent the value added flows calculated as described above. More specifically, the weights $w_{j\to i\left[\nu \right]}$ are the sum of the value added supplied from all sectors of country *j* to the examined sector ν of country *i*, divided by the sum of all transactions among countries, following previously related work [26]. As we are interested in the exchanged foreign value added among countries, the diagonal elements (self-loops) of the weight matrices are set to zero, because each diagonal element represents the domestic contribution. We constructed 810 single-layer GVC networks (54 sectors × 15 years) without overlaps. GVC networks from WIOD data can be constructed in alternative ways. For example, multiplex and multilayer GVC networks [26], GVC (single layer) networks where the nodes are the sectors and the edges are the value of goods flows between the sectors [13]. The selection of the graph model for the analysis of data depends on the goal of the study. In our case, in order to address the three research questions (Section 1 Introduction) we modeled the GVC networks putting the countries as nodes and the flows as edges, following [11].

## 3. Relevant Concepts from Network Theory

We present the relevant concepts from network theory that are used in our analysis.

**Definition** **1** **(** **Weighted** **In-** **and** **Out-Degree).** *The total of the shares of the flows to country i is the weighted in-degree and the total of the shares of the flows from the country i is the weighted out-degree. The weighted degree [35] for each sector ν and each country i = 1, 2, …, 44, are:*

(1) $$deg_{i}^{\left[\nu \right]in}=\overset{44}{\underset{j=1}{\sum }}w_{j\to i\left[\nu \right]},\;deg_{i}^{\left[\nu \right]out}=\overset{44}{\underset{j=1}{\sum }}w_{i\to j\left[\nu \right]}$$

*For each node*i, *we capture the weighted in-degree and weighted out-degree 54-dimensional vectors:*

(2) $$\left(\begin{array}{c} deg_{i}^{\left[1\right]in}\\ \vdots \\ deg_{i}^{\left[54\right]in} \end{array}\right),\;\left(\begin{array}{c} deg_{i}^{\left[1\right]out}\\ \vdots \\ deg_{i}^{\left[54\right]out} \end{array}\right)$$

**Remark** **1.** *The weighted degrees (1) measure the dominance of the country i in the sectoral GVC network, since they were calculated for the value added shares over the global value added flows of sector v. As the weights* $w_{j\to i\left[\nu \right]}$ *represent the input value added share, the country i with the highest weighted in-degree is the dominant importer. The country i with the highest weighted out-degree is the dominant exporter.*

**Definition** **2** **(In-** **and** **Out-Weight** **Entropy).** *The in-weight entropy of node (country)* i=1,2,…,44 *is:*

(3) $$\begin{array}{c} S_{i}^{\left[\nu \right]in}=-\overset{N=44}{\underset{j=1}{\sum }}\rho_{j\to i\left[\nu \right]}^{in}\cdot {log}_{2}\left(\rho_{j\to i\left[\nu \right]}^{in}\right),\;with\;values\;0\leq S_{i}^{\left[\nu \right]in}\leq log_{2}\left(N-1\right)\\ =log_{2}\left(43\right)≃5.43 \end{array}$$

*where:*

(4) $$\rho_{j\to i\left[\nu \right]}^{in}=\frac{w_{j\to i\left[\nu \right]}}{\sum_{j^{\prime }=1}^{44}w_{j^{\prime }\to i\left[\nu \right]}}$$

*is the distribution of the incoming weights of node* i *for each sector* ν *.*

*The out-entropy of node* i=1,2,…,44 *is:*

(5) $$\begin{array}{c} S_{i}^{\left[\nu \right]out}=-\overset{N=44}{\underset{j=1}{\sum }}\rho_{i\to j\left[\nu \right]}^{out}\cdot {log}_{2}\left(\rho_{i\to j\left[\nu \right]}^{out}\right),\;with\;values\;0\leq S_{i}^{\left[\nu \right]out}\leq log_{2}\left(N-1\right)\\ =log_{2}\left(43\right)≃5.43 \end{array}$$

*where:*

(6) $$\rho_{i\to j\left[\nu \right]}^{out}=\frac{w_{i\to j\left[\nu \right]}}{\sum_{i^{\prime }=1}^{44}w_{i^{\prime }\to j\left[\nu \right]}}$$

*is the distribution of the outgoing weights of node* i *for each sector* ν *.*

*If an economy has equally distributed incoming flows from all other economies, the in-entropy receives its maximum value (* ${log}_{2}\left(43\right),$ $w_{i\to i\left[\nu \right]}$ *= 0). Otherwise, if the incoming flows of an economy from its trade partners are fully specialized (imports from one economy), the in-entropy receives its minimum value (zero). If the outgoing flows from an economy to all other economies are equally distributed, the out-entropy receives its maximum value. Otherwise, if the outgoing flows from an economy to the other economies are fully specialized (exports to only one country), the out-entropy receives its minimum value (zero).*

*The normalized entropies are:*

(7) $$J_{i}^{\left[\nu \right]in}=\frac{S_{i}^{\left[\nu \right]in}}{log_{2}\left(N-1\right)}≃\frac{S_{i}^{\left[\nu \right]in}}{5.43},\;J_{i}^{\left[\nu \right]out}=\frac{S_{i}^{\left[\nu \right]out}}{log_{2}\left(N-1\right)}≃\frac{S_{i}^{\left[\nu \right]out}}{5.43}$$

*For each economy* i *, we take the normalized in-entropy and out-entropy 54-dimensional vectors:*

(8) $$\left(\begin{array}{c} J_{i}^{\left[1\right]in}\\ \vdots \\ J_{i}^{\left[54\right]in} \end{array}\right),\;\left(\begin{array}{c} J_{i}^{\left[1\right]out}\\ \vdots \\ J_{i}^{\left[54\right]out} \end{array}\right)$$

**Remark** **2.** *The entropy measure is a neat way of capturing the distribution of flows. The entropies (8) assess the diversification of the incoming and outgoing weights for each sector ν, respectively. Economies with high normalized in-entropy have the most diversified import sources and value-flows. Besides, economies with high normalized out-entropy are exporters with diversified destinations and value flows.*

**Definition** **3** **(In-** **and** **Out-Weighte** **Degree** **Centralization).** *The in-weighted degree centralization of a sectoral GVC network is:*

(9) $$DEG^{\left[v\right]in}=\frac{\sum_{i=1}^{N}\left(deg_{g}^{\left[\nu \right]in}-deg_{i}^{\left[\nu \right]in}\right)}{N-2}$$

*where:*

(10) $$deg_{g}^{\left[\nu \right]in}=\underset{i=1,2,\ldots ,N}{max}\left\{deg_{i}^{\left[\nu \right]in}\right\}$$

*is the degree of the node g with the maximal weighted in-degree for each sector* ν *.*

*The out-weighted degree centralization of a sectoral GVC network is:*

(11) $$DEG^{\left[v\right]out}=\frac{\sum_{i=1}^{N}\left(deg_{g}^{\left[\nu \right]out}-deg_{i}^{\left[\nu \right]out}\right)}{N-2}$$

*where:*

(12) $$deg_{g}^{\left[\nu \right]out}=\underset{i=1,2,\ldots ,N}{max}\left\{deg_{i}^{\left[\nu \right]out}\right\}$$

*is the degree of the node g with the maximal weighted out-degree for each sector* ν *.*

**Remark** **3.** *Centralization counts how dominant the most central nodes are [36]. A centralized network has most of its high weights dispersed around a certain few central nodes. Hence, a centralized sectoral GVC network describes a sector with low competitive conditions that could be characterized as a monopolistic or oligopolistic market. On the other hand, a decentralized sectoral GVC network is highly competitive, as its weights are distributed evenly to most nodes. The centralizations (9), (11) assess the competitive condition with regard to importers and exporters for each sector ν, respectively.*

## 4. Empirical Results and Discussion

### 4.1. Centralization of Sectoral GVC Networks

We use in- and out-weighted degree centralization in order to detect the most and least centralized sectoral GVC networks. We assumed lower competitive conditions in the most centralized sectoral GVC networks as a few countries trade the biggest share of value added of the network; otherwise, we assumed higher competitiveness in the least centralized sectoral GVC network as a few participants trade the biggest share of the value added of the network.

**Figure 1:**
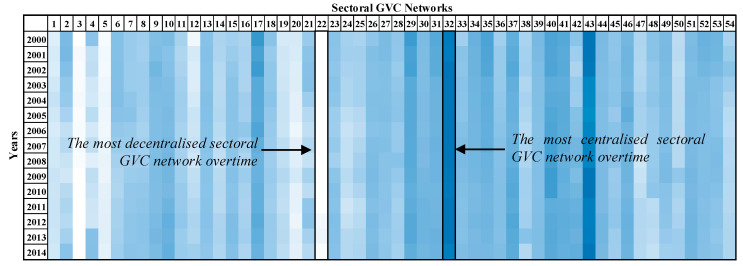
In-weighted degree centralization values evolution of the sectoral global value chains (GVCs) networks through color plot visualization. Columns present the sector and the lines present the year. The more centralized a sectoral GVC network is on the import side, the more of a blue shade it takes on. The more decentralized a sectoral GVC network is on the import side, the more white it is in color. Sectors’ nomenclature is presented in Appendix A.

The results of the in-weighted degree centralization for all of the sectoral GVC networks of the examined period are presented in Figure 1. The most centralized sectoral GVC network, “32—Water transport”, and the least centralized one, the “22—Manufacture of furniture; other manufacturing activities”, are highlighted. The sectors’ nomenclature is presented in Appendix A.

The GVC network of sector “32—Water transport” has the highest in-weighted degree centralization on average (2000–2014) compared with the rest of sectoral GVC networks, few importers are expected to receive a high share of value added from the exporters. On the other hand, the GVC network of sector “22—Manufacture of furniture; other manufacturing activities” has the lowest in-weighted degree centralization on average (2000–2014) compared with the rest of the sectoral GVC networks, therefore the imported share is expected to be uniformly distributed to the importers.

**Figure 2:**
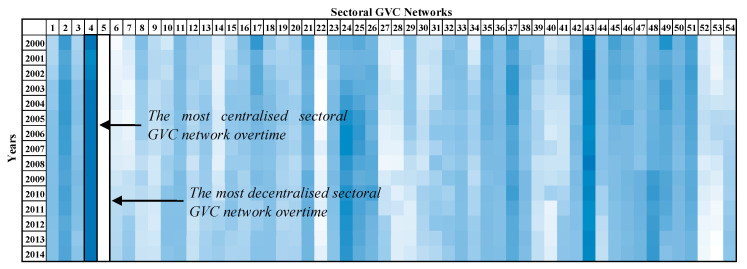
Out-weighted degree centralization values evolution of sectoral GVC networks through color plot visualization. Columns present the sector and the lines present the year. The more centralized a sectoral GVC network is on the export side, the more of a blue shade it takes on. The more decentralized a sectoral GVC network is on the export side, the more white it is in color. Sectors’ nomenclature is presented in Appendix A.

The results of the out-weighted degree centralization for all of the sectoral GVC networks of the examined period are presented in Figure 2. The most centralized sectoral GVC network, “4—Mining and quarrying”, and the least centralized one, the “5—Manufacture of food products, beverages and tobacco products”, are highlighted.

The GVC network of the sector “4—Mining and quarrying” has the highest out-weighted degree centralization on average (2000–2014) compared with the rest of sectoral GVC networks, as few exporters are expected to dominate the supply of the value added. On the other hand, the GVC network of sector “5—Manufacture of food products, beverages and tobacco products” has the lowest out-weighted degree centralization on average (2000–2014) compared with the rest of sectoral GVC networks, therefore the suppliers are expected to export uniformly to importers.

### 4.2. Countries with High Diversification in Sectoral GVC Networks

**Figure 3:**
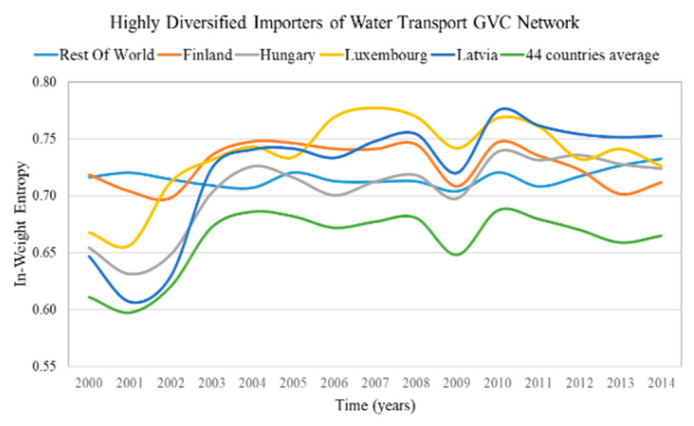
In-weight entropy evolution of countries that rank in the top 5% of each year range of index values (plus the evolution of the 44 countries average value) in the “Water transport” GVC network. The y axis represents the in-weight entropy values and the x axis represents the time.

**Figure 4:**
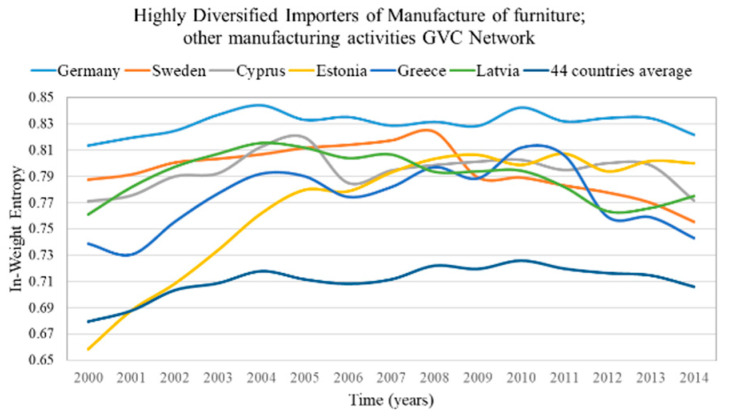
In-weight entropy evolution of countries that rank in the top 5% of each year range of index values (plus the evolution of the 44 countries average value) in the manufacture of furniture; other manufacturing activities GVC network. The y axis represents the in-weight entropy values and the x axis represents the time.

**Figure 5:**
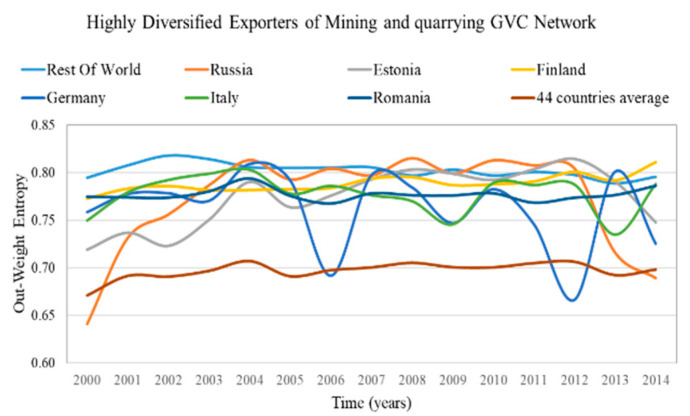
Out-weight entropy evolution of countries that rank in the top 5% of each year range of index values (plus evolution of the 44 countries average value) in the Mining and Quarrying GVC network. The y axis represents the out weight entropy values and the x axis represents the time.

**Figure 6:**
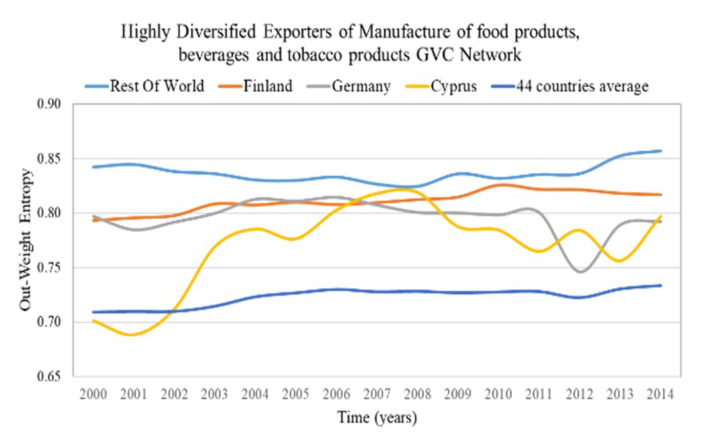
Out-Weight Entropy evolution of countries that rank in the top 5% of each year range of index values (plus evolution of the 44 countries average value) in the “Manufacture of food products, beverages and tobacco products” GVC network. The y axis represents the out-weight entropy values and the x axis represents the time.

The highly diversified countries are characterized by high in-weight entropy (diversified importers) and high out-weight entropy (diversified exporters). We consider as highly diversified importers and exporters the countries with in-weight entropy and out-weight entropy in the top 5%, correspondingly. The results of highly diversified importers and the average value for the most centralized network are presented in Figure 3 and for the most decentralized network in Figure 4. The results of highly diversified exporters and the average value for the most centralized network are presented in Figure 5 and for the most decentralized network in Figure 6.

**Remark** **4.** *A few countries are protected of the low competitive conditions.*

Only a few countries (5–7 of the 44 countries) are highly diversified (have high entropy) in highly centralized networks (Figure 3 and Figure 5). Therefore, in case of changes in the trade conditions of the centralized sectoral GVC networks, the highly diversified countries are expected not to be significantly affected, namely:

- Rest of the world, Finland, Hungary, Luxembourg and Latvia being the highly diversified importers in the GVC network “Water transport”;
- Rest of the world, Russia, Estonia, Finland, Germany, Italy and Romania being the highly diversified exporters in the GVC network “Mining and quarrying”.

**Remark** **5.** *A few countries trade uniformly their share in the decentralized networks.*

In the sectoral GVC networks with the lowest centralization (Figure 4 and Figure 6), only a few countries (4–6 of the 44 countries) are highly diversified (have high entropy). This is surprising because one would expect from Remark 4 more highly diversified countries in decentralized networks. These highly diversified countries are:

- Germany, Sweden, Cyprus, Estonia, Greece and Latvia are the highly diversified importers in the GVC network “Manufacture of furniture; other manufacturing activities”;
- Rest of the world, Finland, Germany and Cyprus are the highly diversified exporters in the GVC network “Manufacture of food products, beverages and tobacco products”.

**Remark** **6.** *Diversification (high entropy) is not conditioned by competitiveness (low centralization).*

From the in- and out-weight entropy range of the centralized and decentralized sectoral GVC networks (Figure 3, Figure 4, Figure 5 and Figure 6), we observe that the entropy of highly diversified countries moves within a similar range (0.64–0.86). Consequently, diversification does not depend on centralization and vice versa, as one would expect from Remark 4.

**Remark** **7.** *Input–output balance does not prevent domino effects.*

The average in-weight entropy and out-weight entropy are approximately equal (0.60–0.73), Figure 3, Figure 4, Figure 5 and Figure 6. Therefore, importing shares on the average keep up with exporting shares. This approximate balance indicates that domino effects in sectoral GVC networks in case of a country slowdown–collapse are not prevented or controlled by default. Policy interventions are required. Further analysis of long-range economic connections between countries sheds light on crisis contagion as cascade effect [37].

### 4.3. Countries with Dominant Position in the Sectoral GVC Networks

**Figure 7:**
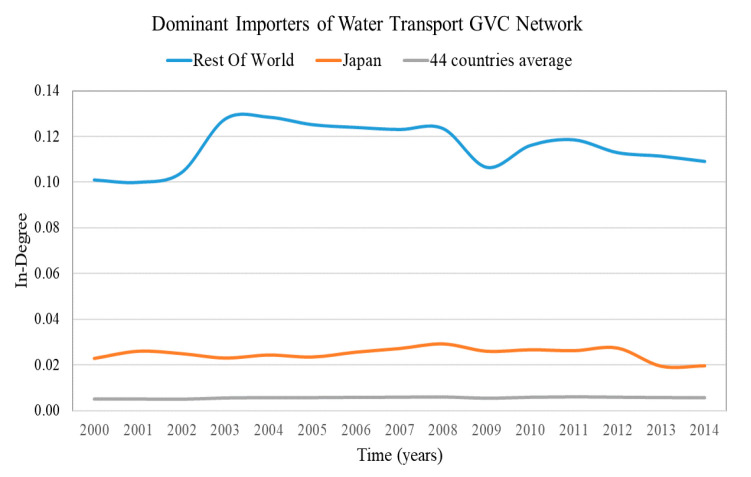
Weighted in-degree evolution of countries that rank in the top 5% of each year range of index values (plus the evolution of the 44 countries’ average value) in the “Water transport” GVC network. The y axis represents the weighted in-degree values and the x axis represents the time.

**Figure 8:**
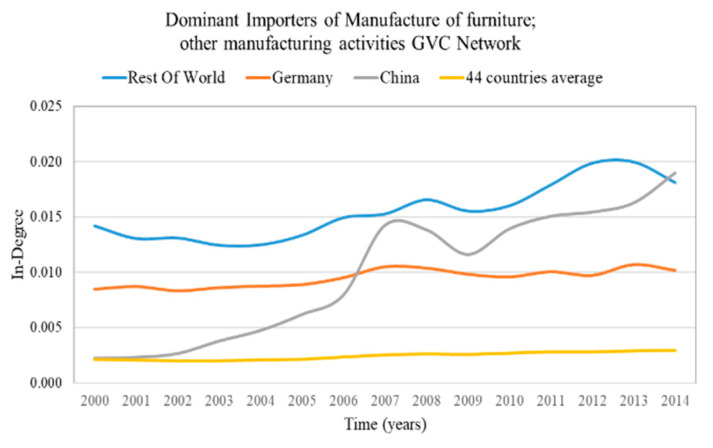
Weighted in-degree evolution of countries that rank in the top 5% of each year range of index values (plus the evolution of the 44 countries’ average value) in the “Manufacture of furniture; other manufacturing activities” GVC network. The y axis represents the weighted in-degree values and the x axis represents the time.

**Figure 9:**
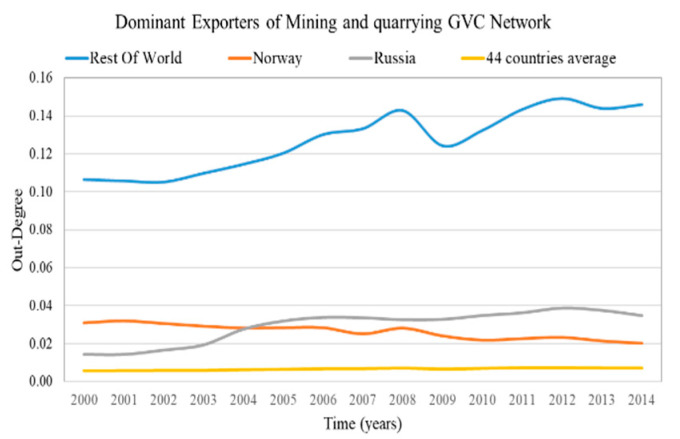
Weighted out-degree evolution of countries that rank in the top 5% of each year range of index values (plus the evolution of the 44 countries’ average value) in the “Mining and quarrying” GVC network. The y axis represents the weighted out-degree values and the x axis represents the time.

**Figure 10:**
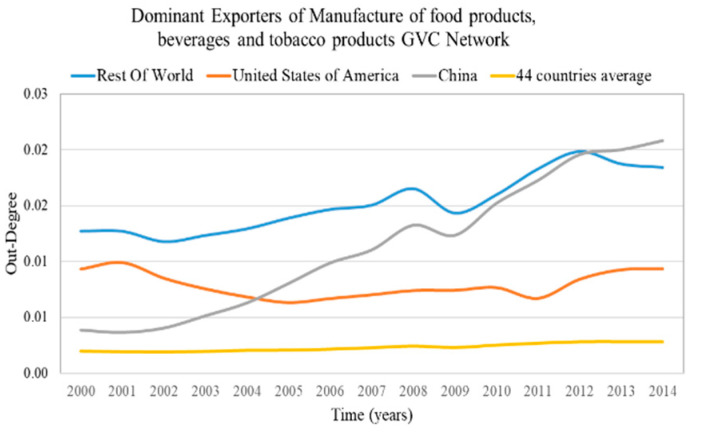
Weighted out-degree evolution of countries that rank in the top 5% of each year range of index values (plus the evolution of the 44 countries’ average value) in the “Manufacture of food products, beverages and tobacco products” GVC network. The y axis represents the weighted out-degree values and the x axis represents the time.

The countries with a dominant position are characterized by high weighted in-degree (dominant importers) and high weighted out-degree (dominant exporters). We consider as dominant importers and exporters the countries with weighted in-degree and weighted out-degree in the top 5%, correspondingly. The results of dominant importers and the average value of the most centralized network are presented in Figure 7 and for the most decentralized network are presented in Figure 8. The results of dominant exporters and the average value of the most centralized network are presented in Figure 9 and for the most decentralized network are presented in Figure 10.

**Remark** **8.** *Dominant countries (rich) become more dominant (richer).*

Only a few countries (2–3 of the 44 countries) are dominant (have high degree) both in centralized and decentralized sectoral GVC networks (Figure 7, Figure 8, Figure 9 and Figure 10). Moreover, the dominant countries increase their shares while the average value remains approximately constant. Therefore, as the dominant countries increase their shares, the other countries lose their shares. This is a manifestation of the Matthew effect “the rich get richer and the poor get poorer” [38] in the sectoral GVC networks. These dominant countries are:

- Rest of the world and Japan are the dominant importers in the highly centralized GVC network “Water transport”;
- Rest of the world, Norway and Russia are the dominant exporters in the highly centralized GVC network “Mining and quarrying”;
- Rest of the world, Germany and China are the dominant importers in the decentralized GVC network “Manufacture of furniture; other manufacturing activities”;

Rest of the world, the United States of America and China are the dominant exporters in the decentralized GVC network “Manufacture of food products, beverages and tobacco products”.

**Remark** **9.** *The leading dominant country is not affected in networks with low competition (high centralization), while vulnerable in networks with high competition (low centralization).*

The leading rest of the world remains most dominant in the centralized sectoral GVC networks (Figure 7 and Figure 9), while in the decentralized sectoral GVC networks (Figure 8 and Figure 10), China, initially in third place, becomes the leading dominant country. China has emerged as the “World Factory” [39] in many sectoral GVC networks.

**Remark** **10.** *Diversification (entropy) and dominance (degree) are not correlated.*

**Figure 11:**
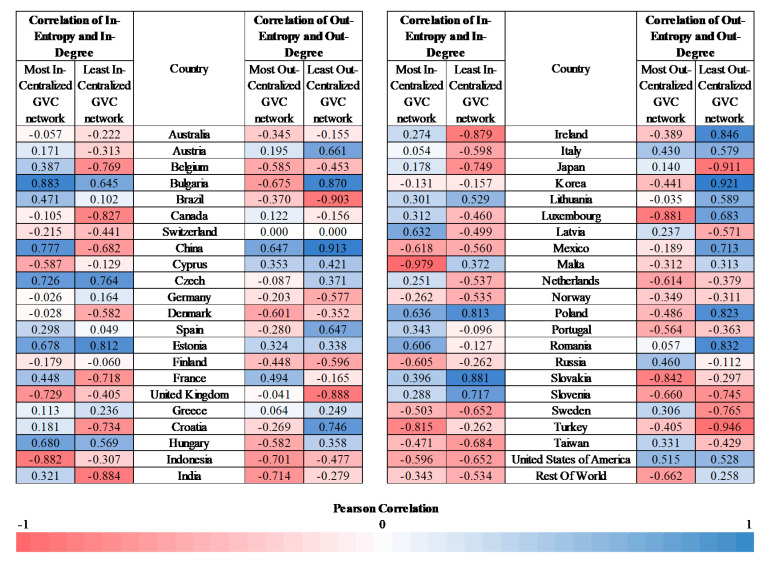
Correlation of entropy and the degree of each country of the most and least centralized sectoral GVC networks for the period 2000–2014.

There is no correlation between dominance (degree) and diversification (entropy), as shown by the computation of the Pearson Correlation in Figure 11. In the most (in/out) centralized GVC networks, the cross-country average correlation of in-entropy and in-degree is 0.05 and the cross-country average correlation of out-entropy and out-degree is −0.18. In the least (in/out) centralized GVC network, the cross-country average correlation of in-entropy and in-degree is −0.20 and the cross-country average correlation of out-entropy and out-degree is −0.04. Although the leading dominant country is also highly diversified, the other dominant countries are not necessarily highly diversified (Figure 3, Figure 5, Figure 6, Figure 7, Figure 8, Figure 9 and Figure 10). As the dominant United States of America, China, Japan and Norway are not highly diversified, they are more vulnerable to shocks. Countries with low entropy are highly specialized regarding their trade partners [24,25]. In this case, a shock or a slowdown in a partner’s economy may have significant effects on trade between them and also on the country’s economy. Further examination of the vulnerability to shocks requires an analysis of the interactions in cascade between the countries [37].

**Remark** **11.** *The developing economies are collectively diversified and dominant.*

The rest of the world represents collectively developing countries, which are both diversified (entropy, Figure 3, Figure 5 and Figure 6) and dominants (degree, Figure 7, Figure 8, Figure 9 and Figure 10). Moreover, the dominance of the rest of the world appeared to increase in time (Figure 7, Figure 8, Figure 9 and Figure 10). This is an emerging self-organized strategy with high expectation for developing and third world economies in the global trade network.

## 5. Concluding Remarks

We investigated the diversification, the competitiveness and the dominance of sectoral GVC networks, using entropy, centralization and network topology from the data from the period 2000–2014. More specifically, after constructing the sectoral GVC networks, we assessed: (a) the most diversified participants; (b) the degree of centralization of the sectoral GVC networks; and (c) the dominant countries.

The key findings of our work are summarized as follows:

Research question 1: Which sectoral GVC networks are highly centralized and what is the extent of centralization? Our study reveals that centralization varies widely in sectoral GVC networks (Section 4.1). Centralization proved to be a useful tool to directly assess the competitive conditions in sectoral GVC networks, completing previous theoretical and empirical results [9]. On the imports side, the most centralized network is “Water transport” while the most decentralized is the “Manufacture of furniture; other manufacturing activities”. On the exports side, the most centralized network is the “Mining and quarrying” while the most decentralized is the “Manufacture of food products, beverages and tobacco products”.

Research question 2: Which countries within a sectoral GVC network are highly diversified? While one would expect more countries to be diversified in decentralized networks, we found that diversification is not conditioned by competitiveness (Remark 6). Only a few certain countries are highly diversified in centralized and decentralized networks (Remark 4–5): the rest of the world, Finland, Hungary, Luxembourg and Latvia are the most diversified importers and rest of the world, Russia, Estonia, Finland, Germany, Italy and Romania are the most diversified exporters of the corresponding centralized sectoral GVC networks. On the other hand, Germany, Sweden, Cyprus, Estonia, Greece and Latvia are the most diversified importers and the rest of the world, Finland, Germany and Cyprus are the most diversified exporters of the corresponding decentralized sectoral GVC networks. Furthermore, the presence of input–output balance does not prevent domino effects in sectoral GVC networks in case of a country slowdown–collapse (Remark 7).

Research question 3: Are the dominant countries within a sectoral GVC network also highly diversified? Although the leading dominant country is also highly diversified, the other dominant countries are not necessarily highly diversified (Remark 10). The developing economies are collectively diversified and dominant (Remark 11). Dominants countries increase their shares while the average value remains stable. This allows dominant countries to expand their share at the expense of the rest (Remark 8). Moreover, the leading dominant country is not affected in networks with low competition, while vulnerable in networks with high competition (Remark 9).

Three major findings emerge from our research, each with its own policy implications. First, countries wishing to enter or increase its participation in global trade should implement more concentrated trade policies on less centralized sectoral networks, as they have more chance (Remark 9, Figure 8 and Figure 10). Second, a dominant country is not necessarily diversified, but when it does, it is difficult to lose its dominance (Figure 3, Figure 5, Figure 7 and Figure 9). Third, developing economies adopting diversification strategies have the chance to improve their dominance in the GVC networks (Remark 11).

We confirmed that GVCs are becoming more consolidated [9]. The world production and trade become distributed, structured in the form of GVCs at an increasing level. The emergence and evolution of supply chains [4,5,6,30,40,41,42,43,44,45,46] helped most countries to be integrated into the global economy. Our contribution in this direction is the demonstration that in the sectoral GVC networks the rich (dominant) become richer (more dominant), pushing competition (Remark 8).

Our goal is to highlight the competition in sectoral GVC networks in terms of a first exploratory network analysis. In this direction, we used the degree of centralization as a preliminary tool for the exploration of the evolution of competition in sectoral GVC networks, following previous theoretical and empirical results [9]. Further detailed analysis using finer tools of network theory, like PageRank, CheiRank, HITS (Hubs–Authorities) and other centrality measures will be the subject of a subsequent paper. Using entropy and network analysis, we can study network resilience in shocks, and address questions like “too big to fail?” or “too smartly interconnected to fail?”. In this preliminary work, we analyzed the global value chain in terms of 54 different sectoral networks with countries as nodes and edges weighted by the sum of all transactions among countries (end of Section 2). Therefore, our work is limited, because we do not asses the flows among sectors. This important question requires the analysis of the multigraph of 54 different sectoral networks. The multigraph analysis of GVC networks has already been initiated [26]. We intend to consider the multigraph analysis in the future.

## Appendix A

Sectors’ nomenclature in WIOD2016R	
No.	Sector name
1	Crop and animal production, hunting and related service activities
2	Forestry and logging
3	Fishing and aquaculture
4	Mining and quarrying
5	Manufacture of food products, beverages and tobacco products
6	Manufacture of textiles, wearing apparel and leather products
7	Manufacture of wood and of products of wood and cork, except furniture; manufacture of articles of straw and plaiting materials
8	Manufacture of paper and paper products
9	Printing and reproduction of recorded media
10	Manufacture of coke and refined petroleum products
11	Manufacture of chemicals and chemical products
12	Manufacture of basic pharmaceutical products and pharmaceutical preparations
13	Manufacture of rubber and plastic products
14	Manufacture of other non-metallic mineral products
15	Manufacture of basic metals
16	Manufacture of fabricated metal products, except machinery and equipment
17	Manufacture of computer, electronic and optical products
18	Manufacture of electrical equipment
19	Manufacture of machinery and equipment n.e.c.
20	Manufacture of motor vehicles, trailers and semi-trailers
21	Manufacture of other transport equipment
22	Manufacture of furniture; other manufacturing activities
23	Repair and installation of machinery and equipment
24	Electricity, gas, steam and air conditioning supply
25	Water collection, treatment and supply
26	Sewerage; waste collection, treatment and disposal activities; materials recovery; remediation activities and other waste management services
27	Construction
28	Wholesale and retail trade and repair of motor vehicles and motorcycles
29	Wholesale trade, except of motor vehicles and motorcycles
30	Retail trade, except of motor vehicles and motorcycles
31	Land transport and transport via pipelines
32	Water transport
33	Air transport
34	Warehousing and support activities for transportation
35	Postal and courier activities
36	Accommodation and food service activities
37	Publishing activities
38	Motion picture, video and television program production, sound recording and music publishing activities; programming and broadcasting activities
39	Telecommunications
40	Computer programming, consultancy and related activities; information service activities
41	Financial service activities, except insurance and pension funding
42	Insurance, reinsurance and pension funding, except compulsory social security
43	Activities auxiliary to financial services and insurance activities
44	Real estate activities
45	Legal and accounting activities; activities of head offices; management consultancy activities
46	Architectural and engineering activities; technical testing and analysis
47	Scientific research and development
48	Advertising and market research
49	Other professional, scientific and technical activities; veterinary activities
50	Administrative and support service activities
51	Public administration and defense; compulsory social security
52	Education
53	Human health and social work activities
54	Other service activities
